# Spatiotemporal patterns and drivers of soil organic carbon in black soil landscapes of Northeast China

**DOI:** 10.1371/journal.pone.0320784

**Published:** 2025-06-02

**Authors:** Kai Liu, Yunhong Song, Shouying Du, Hongye Xiao, Chaoqun Chen, Jiang Xu, Huimin Dai, Nana Fang

**Affiliations:** 1 Key Laboratory of Black Soil Evolution and Ecological Effect, Ministry of Natural Resources/Liaoning Province, Shenyang, Liaoning, China; 2 Shenyang Center of China Geological Survey, Northeast Geological S&T Innovation Center of China Geological Survey, Shenyang, Liaoning, China; 3 Shenyang Pengde Environmental Technology Co., Ltd., Shenyang, Liaoning, China; Zhejiang Agriculture and Forestry University: Zhejiang A and F University, CHINA

## Abstract

Soil Organic Carbon (SOC) is crucial for soil health, agricultural productivity, and climate regulation. This study examines the temporal and spatial changes in SOC over a decade (2013–2023) in the Tongken River Basin, a key area within the black soil region of Northeast China. Using machine learning techniques and advanced spatial mapping techniques, the study identified temperature, precipitation, and soil managements as key drivers of SOC dynamics. The results revealed a significant increase in SOC content from 2.99% to 3.25%, and SOC density rose from 7.08 kg/m² to 7.72 kg/m², with precipitation exerting the strongest positive influence. These findings highlight the potential of climate-smart land-use strategies to enhance SOC storage and mitigate soil degradation. This research provides valuable insights for sustainable soil management and climate adaptation efforts in vulnerable agricultural regions.

## 1. Introduction

Soil Organic Carbon (SOC) plays a crucial role in maintaining soil fertility, which is essential for crop productivity, while also acting as a carbon sink to mitigate global warming [1,2]. Given the importance of SOC for both agriculture and climate regulation, understanding its spatiotemporal dynamics is critical. Despite prior research, potential gaps remain in linking these changes to key climatic and land-use factors, particularly in the context of specific regions like the black soil area in Northeast China.

The dynamics of SOC are shaped by a variety of factors, including climatic conditions and human activities [3]. Temperature and precipitation patterns are significant climatic determinants that affect SOC decomposition and accumulation [4]. Rising temperature trends expedite SOC decomposition [5], while changes in precipitation patterns impact soil moisture and microbial activity, which in turn affect the soil’s carbon content [6]. Furthermore, human activities—such as land use changes and agricultural practices—can either enhance or deplete SOC levels. For example, the conversion of cropland to forest or grassland generally increases SOC, while intensive farming practices, like continuous cropping, can lead to SOC depletion [7,8].

Although studies in regions like the Midwestern United States and Europe have explored the impact of climate and land-use changes on SOC[9–11], similar research in Northeast China’s Black Soil Region remains limited. The region is critical for China’s grain production but has faced declining SOC levels due to intensive agricultural practices and climate change [8,12]. Long-term overcultivation and inadequate agricultural practices have been identified as the primary causes of this decline [13,14], with SOC levels dropping since the 1980s, making it the only region in China to experience such a trend [15]. This decline threatens soil quality and agricultural sustainability.

In response, the Chinese government launched the Northeast Black Land Protection Project [16], promoting agro-ecological practices like straw returning and conservation tillage [17]. Conventional farming techniques, such as plowing and deep tillage, typically induce the oxidation and breakdown of soil organic matter, thereby diminishing the SOC content [18]. In contrast, conservation agriculture and vegetative cover contribute to the mitigation of SOC loss and foster the accumulation of organic matter[19–21]. Notably, under sustained vegetative cover, the retention of organic materials in the soil is more probable, thereby preserving or augmenting SOC content [22]. These strategies have shown promise in increasing SOC content [23,24]. However, there is ongoing debate about the sustainability of SOC increases, particularly in the context of changing climatic conditions. While some studies suggest that improved land management can boost SOC[7,20,25,26], others highlight the challenges posed by climate variability [4].

While much research has focused on the general impacts of climate and land use on SOC, few studies have examined the spatiotemporal distribution patterns of SOC in Northeast China’s Black Soil Region and the specific climatic and land-use drivers behind these patterns [27]. This study aims to address these gaps by focusing on the Tongken River Basin in Northeast China, an area where conservation tillage has been widely adopted [16,25]. Using soil samples from 2013 and 2023, we construct a spatiotemporal distribution map of SOC through Kriging interpolation, a method that ensures reliable estimation of spatial variability while providing error data for validation. Additionally, a random forest model is employed to identify key environmental factors driving SOC variations.

This study has two primary objectives: (1) to characterize the spatiotemporal distribution model of SOC in the Black Soil Region, and (2) to quantify the key environmental factors regulating SOC distribution. The findings will contribute to a better understanding of SOC dynamics in this region and offer insights for sustainable soil management strategies.

## 2. Materials and methods

### 2.1 Study area

**Fig 1:**
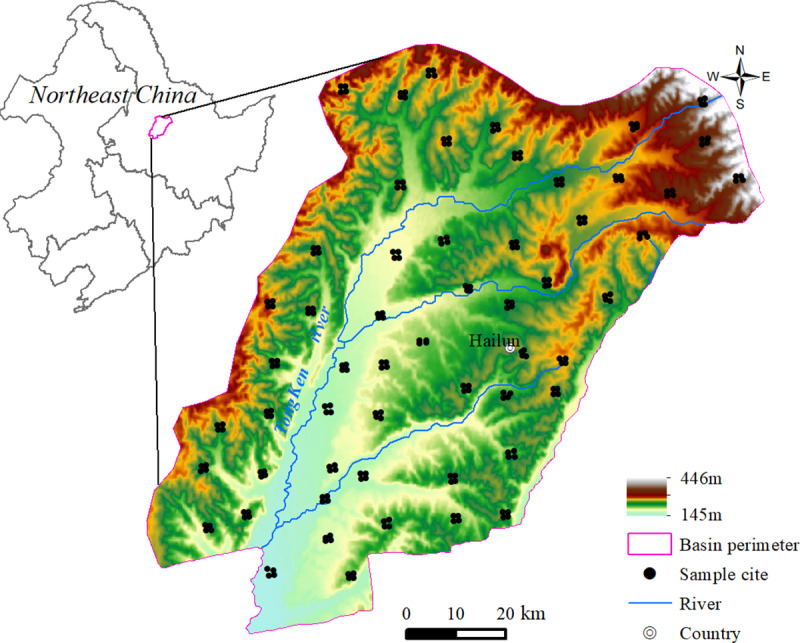
The elevation of Tongken river basin and distribution of the sampling cites.

**Fig 2:**
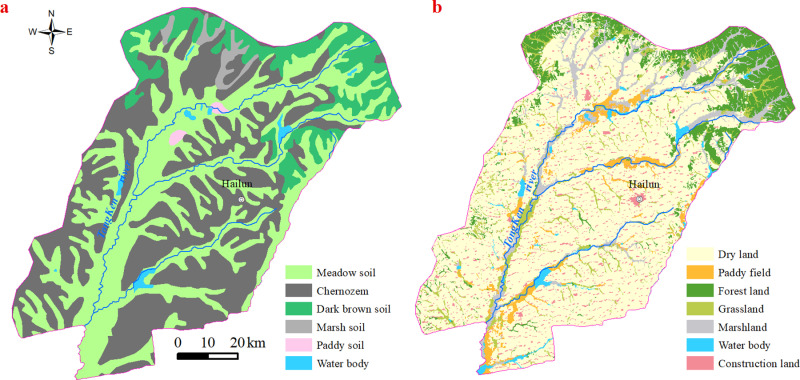
(a) The soil types in Tongken basin; (b) The land use types in Tongken basin.

The Tongken River Basin is situated in the north-central part of northeast China and covers an area of 7,910 square kilometers (Fig 1). The topography predominantly consists of plains, with hilly terrain towards the northeast, characterized by elevations ranging from 145 to 446 m. It experiences a continental monsoon climate with a short and warm rainy summer and long, cold, dry winter. The average annual temperature ranges from 2 to 4.4°C, gradually decreasing from south to north. The annual average precipitation varies between 570–660 mm, gradually decreases from east to west. In the hilly region, the predominant soil type is dark brown soil covering an area of 914.2 km²; whereas in the high plain areas it mainly comprises black soil spanning over an area of 3,297.5 km². Meadow soil was distributed in the lower plains on both sides of the river, occupying an area of 3,358.1 km² (Fig 2). The land use pattern within this study area primarily consists of dry land accounting for approximately 5,362.13 km² (67.79%), mainly utilized for cultivating corn and soybean crops; forest land covers around 859.66 km² (10.87%); marshland occupies about 6.82%; grassland accounts for approximately 5.65%; construction land encompasses roughly 3.71%; paddy fields cover about 3.61%, while water covers approximately 1.55% (Fig 2). Hailun City in Heilongjiang Province serves as the primary administrative region within this research area and has been designated as a demonstration zone for straw return practices in China [24,25].

### 2.2 Soil sampling and testing

The soil samples used in this study were collected in 2013 and 2023, with a one-to-one correspondence between the two sampling years. Sampling was carried out in the spring (April) to capture seasonal variations, with samples collected from the two primary ecosystems: dryland and forest. Soil samples were obtained from depths of 0–20 cm, with each sampling point marked consistently at the same location across both years. The collected topsoil samples were air-dried to eliminate excess moisture, while preserving the soil structure. After drying, the soil samples were ground and screened to remove gravel content. The SOC content test specimens were then passed through a 10-mesh sieve and sent to the laboratory for analysis. The results are expressed as a percentage of the soil dry weight using the potassium dichromate volumetric method. Soil bulk density (BD) was determined using the ring knife method, where the soil sample was extracted and its dry weight was determined.

### 2.3 SOC density calculation

The SOC density calculation (SOCD) involves the analysis of the SOC content, gravel composition (particle size >2 mm), and BD. The SOCD for the top 20 cm layer was calculated using the following formula: SOCD = SOC × BD × H × (1 − G) ×10, where SOCD is the organic carbon density in kg⋅m^-2^, SOC is the organic carbon content in %, BD is the soil bulk density in g⋅cm^−3^, H represents the soil thickness at 20 cm, and G represents the gravel content as a percentage.

### 2.4 Statistical analysis and spatial interpolation techniques

#### 2.4.1 Descriptive statistics.

Descriptive statistics were used to analyze the SOC and SOCD parameters, including measures such as mean, standard deviation, skewness, kurtosis, and other statistical indicators. All statistical analyses were performed using R software (version 3.6.1). The paired t-test, assuming normal distribution of data and equal variances between groups, was used to assess significant differences in SOC content between the two distinct time periods. A significance threshold of p < 0.05 was applied.

#### 2.4.2 Spatial autocorrelation analysis.

Spatial autocorrelation analysis involved the application of the global Moran’s I index to examine interdependencies among variables and determine spatial autocorrelation on a global scale. The global Moran’s I index, calculated using ArcGIS (version 10.7), was used to evaluate accumulation patterns of SOC content.. The index value ranged from -1–1; positive values indicated an accumulation distribution of factors, whereas negative values indicated dispersion. Values approaching zero imply randomness in the distribution, with higher absolute values indicating stronger correlation. Normalized Z-scores were used for significance testing: |Z| > 1.96 was considered significant autocorrelation, while lower values indicated no significant autocorrelation.

#### 2.4.3 Spatial interpolation analysis.

Spatial interpolation analysis was conducted using the GS+ software (version 9.0) to perform a preliminary statistical analysis of the sample data. By calculating the obtained semi-variogram values, different semi-variogram models were fitted and their parameters were calculated. This allowed us to obtain the spatial characteristics of the soil properties through semi-variograms and select the optimal fitting model based on the determination coefficient R^2^ and residual square (RSS). Generally, a larger R^2^ value indicates a smaller RSS value, which represents the optimal model choice. In this study, the spherical, exponential, and Gaussian models were compared and selected. Once the model parameters were determined, Kriging interpolation was applied using ArcGIS (version 10.7), chosen for its ability to provide accurate spatial predictions based on the semi-variogram model. Kriging was deemed suitable for spatially correlated data, providing unbiased predictions with optimal precision.

#### 2.4.4 Spatiotemporal variation error analysis and significance test.

The SOC interpolation grid data (SOC_2013_ and SOC_2023_) and its corresponding standard error data (E_2013_ and E_2023_) for 2013 and 2023, respectively, were derived from the Kriging interpolation results. By applying the formula dSOC = SOC_2023_–SOC_2013_, spatial change data for the SOC content were obtained.

The prediction uncertainty at each interpolation point can be assessed by considering the standard deviation of the estimates generated during the Kriging interpolation process. When calculating this deviation, it is important to account for the error associated with each grid as it propagates through to affect the final result. The standard error for the difference in dSOC was calculated as:


$$\text{dE=}\sqrt{E_{2013}^{2}+E_{2023}^{2}}$$

To assess statistical significance, a t-test was conducted. If |dSOC| > 1.96 * dE, a significant change in SOC content was considered at a 95% confidence level. If -1.96 * dE ≤ dSOC ≤ 1.96 * dE, no statistically significant change was observed.

### 2.5 Random forest model

The random forest (RF) model was employed to determine the driving factors of temporal and spatial changes in SOC content. The RF model is a non-parametric approach comprising numerous individual tree models trained on a self-service sample of data [28]. The optimal number of regression trees and split trees was determined based on the out-of-pocket data errors. The final predicted value was the average of all tree models. This model was chosen for its ability to handle high-dimensional data and its robustness in feature selection. Additionally, the RF model is well-known for its high tolerance to outliers and noise, which helps avoid overfitting and improves generalization to unseen data. Given its strong performance in identifying complex patterns and interactions in ecological data, RF is an ideal choice for modeling the factors influencing SOC changes. In this study, the RF package developed by Liaw and Wiener was used in R software (version 3.6.1), with the number of trees (mtree) set at 500.

The dSOC grid, which represents the change in SOC content, serves as the dependent variable in the RF model. The explanatory variables included trends in temperature, rainfall, normalized plant index, net primary productivity, population density, and land-use changes over the past 10 years.

The performance of the RF model was evaluated by partitioning the total dataset into a training set (70%) and test set (30%). Stability was assessed using R2, with higher values indicating greater stability, whereas accuracy was evaluated using the RMSE, with lower values indicating higher accuracy. Finally, the RF model provided an importance ranking of the independent variables presented as percentages.

### 2.6 Data resources

When investigating the driving forces behind SOC change, three key indicators–climate, vegetation, and human activities–were utilized to assess their influence on SOC dynamics (Table 1).

**Table 1:** Driving factors of SOC dynamic used in this study.

Indicator type	indicator	Year range	Data Resolution	Data source
Climate	Mean Annual Precipitation (MAP)	10 years, 2013–2022	1km×1km	National Tibetan Plateau Data Center (https://data.tpdc.ac.cn)
	Mean Annual Temperature (MAT)	10 years, 2013–2022	1km×1km	National Tibetan Plateau Data Center (https://data.tpdc.ac.cn)
Vegetation	Net primary productivity (NPP)	10 years, 2013–2022	500m×500m	Geographic Data Sharing Infrastructure, global resources data cloud (www.gis5g.com)
	Normalized difference vegetation index (NDVI)	10 years, 2013–2022	250m×250m	National Tibetan Plateau Data Center (https://data.tpdc.ac.cn)
Human activity	Land use	2 years, 2013 and 2022	30m×30m	Resources and Environmental Science and Data Center of the Chinese Academy of Sciences (www.resdc.cn)
	Population density (POP)	10 years, 2013–2022	1km×1km	Https://landscan.ornl.gov

#### 2.6.1 Climate indicators.

Temperature and precipitation are crucial climate indicators that impact the dynamics of SOC. The annual mean temperature and rainfall datasets from 2013 to 2022 with a grid resolution of 1 km were collected using the delta spatial downscaling scheme in China based on the global 0.5° climate dataset released by CRU and the global high-resolution climate dataset released by WorldClim [29]. The data were validated using 496 independent meteorological observation points. The data were obtained from the National Tibetan Plateau Data Center (TPDC).

#### 2.6.2 Vegetation indicators.

The normalized difference vegetation index (NDVI) and net primary productivity (NPP) were used as indicators of vegetation growth during different periods. NDVI data were derived from the Aqua/Terra-MODIS satellite sensor MOD13Q1 product and land-use data [30] obtained at a spatial resolution of 250m from the National Tibetan Plateau Data Center (TPDC). NPP data, sourced from the Modis satellite remote sensing information, were generated using the GEE platform with a spatial resolution of 500m for the years 2013–2022, referencing both the Biome-BGC model and light energy utilization model.

#### 2.6.3 Human activity indicators.

The population density can be used to indicate the level of human activity. The data source utilized in this study was LandScan global population distribution raster data, which was developed by the Oak Ridge National Laboratory (ORNL) under the Department of Energy (DOE). The spatial resolution of the dataset was 1 km.

This study utilized the multi-period land use remote sensing monitoring dataset of China as the source for land use data, with Landsat remote sensing images serving as the primary information source and 30-meter grid data constructed through manual visual interpretation. Specifically, this study employed land-use data from 2013 and 2022 obtained from the Resources and Environmental Science and Data Center of the Chinese Academy of Sciences (www.resdc.cn).

#### 2.6.4 Data conversion.

**Fig 3:**
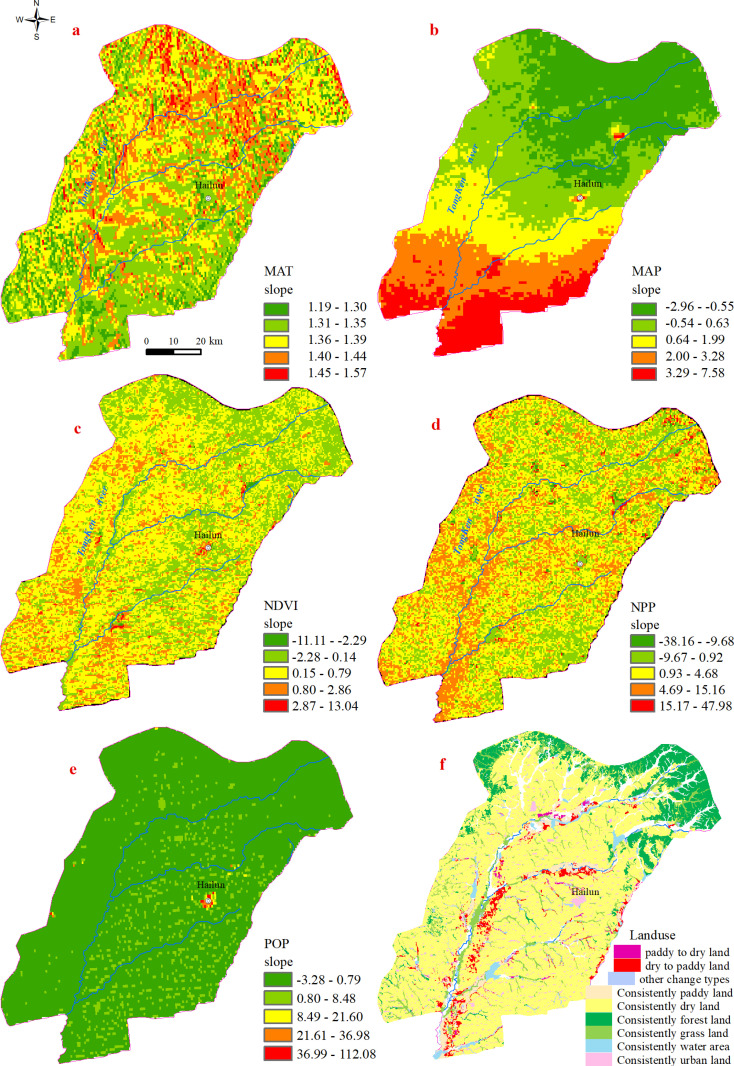
(a) Trends of annual average temperature from 2013 to 2023; (b) Trends of annual precipitation from 2013 to 2023; (c) Trends of NDVI from 2013 to 2023; (d) Trends of NPP from 2013 to 2023; (e) Trends in population density from 2013 to 2023; (f) Changes in land use types from 2013 to 2023.

The above numerical explanatory variables were resampled into 500m grids using ArcGIS, and the regression equation of each grid unit from 2013 to 2022 was calculated using the unary linear regression method. The slope was used to represent the temporal change trend over this period and to generate new grids. A positive slope indicates an overall upward trend in the index over a span of ten years, a zero slope indicates no significant change in the index, and a negative slope indicates an overall downward trend. This trend variable plays a crucial role in modeling changes in organic carbon [31]. The temperature change trend was labeled as TMP_SL, rainfall change trend as PRE_SL, normalized difference vegetation index change trend as NDVI_SL, net primary productivity change trend as NPP_SL, and population density change trend as P0P_SL (Figs 3a–3e). Grid data reflecting land-use changes over a decade were obtained by intersecting land-use data from two time periods in ArcGIS (e.g., consistently dry land or forest land; dryland to paddy land) (Fig 3f).

## 3 Results

### 3.1 Descriptive statistics of SOC

#### 3.1.1 SOC content and SOC density.

The paired t-test results revealed significant differences in the SOC content and SOC density between 2013 and 2023 (p < 0.01). As presented in Table 2, the average SOC content exhibited an increase from 2.99% to 3.25%, accompanied by a rise in SOC density from 7.08 kg/m² to 7.72 kg/m², indicating an average annual carbon sequestration rate of 0.064 kg/m². These findings suggest that the soil in the study area functioned as a carbon sink overall during the period of 2013–2023, aligning with the findings reported by [23]. The increase in SOC content can be associated with the benefits of conservation tillage practices in reducing soil degradation, which could inform land management strategies aimed at enhancing carbon sequestration in agricultural regions. The relatively stable coefficient of variation (0.33–0.39) also indicates moderate spatial heterogeneity, which suggests that soil management practices could help maintain or even enhance this carbon sequestration potential across the landscape.

**Table 2:** Descriptive statistics of SOC content and SOC density.

	Year	Mean	SD	Min	Median	Max	skewness	kurtosis	CV
SOC content (%)	2013	2.99	0.97	1.64	2.76	5.56	0.84	-0.10	0.33
	2023	3.25	1.23	1.40	2.97	8.43	1.65	3.34	0.38
SOC density (kg/m^2^)	2013	7.08	2.32	3.71	6.63	12.58	0.85	-0.12	0.33
	2023	7.72	3.03	3.62	6.97	21.40	1.64	3.42	0.39

SD, standard deviation; Min, minimum; MAX, maximum; CV, coefficient variable.

#### 3.1.2 SOC content of different land use types.

**Fig 4:**
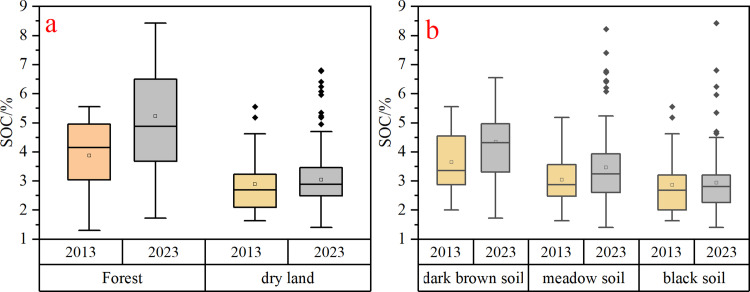
Soil organic carbon (SOC) content in different land use types (a) and different soil types (b).

The primary land use types in the study area were dry land and forest land, accounting for 67.79% and 10.87% of the total area, respectively. Given the small area and limited sample size of other land use types, we compared SOC content between dry land and forest land (Fig 4a). Forest soils exhibited significantly higher SOC content compared to dry fields, with a median SOC content of 4.15% in 2013 and 4.88% in 2023, while dry fields showed SOC content of 2.69% in 2013 and 2.89% in 2023. Over the ten-year period, forest SOC content increased by 17.59%, while dry-field SOC content increased by 10%. These results underscore the importance of forest management practices in carbon sequestration, and they suggest that afforestation and reforestation efforts could be effective strategies for enhancing SOC in agricultural landscapes. Furthermore, the results suggest that conservation practices aimed at preserving forest cover could significantly contribute to climate change mitigation goals.

#### 3.1.3 SOC content of different soil types.

The median SOC content in the dark brown soils was 3.36% in 2013 and increased to 4.32% by 2023. The meadow soils displayed median SOC levels of 2.88% and 3.24% in 2013 and 2023, respectively. In contrast, black soils exhibited median SOC values of 2.68% and 2.81% in 2013 and 2023, respectively. Dark brown soils, prevalent in hilly forested regions, had the highest SOC content, whereas black soils, predominantly found in cultivated areas, had the lowest SOC levels. Overall, there was a noticeable increase in SOC levels across the different soil types (Fig 4b). These findings highlight that soil type plays a significant role in SOC content, which is important for soil management practices. For example, areas with dark brown soils may offer more potential for carbon sequestration, thus influencing land-use planning and climate adaptation strategies in regions with varying soil types (Fig 4b). This spatial variability suggests that targeted soil management practices should be tailored to specific soil types for maximizing SOC storage.

### 3.2 Spatial and temporal distribution of SOC content

#### 3.2.1 Spatial autocorrelation analysis.

Spatial autocorrelation analysis of SOC content across two distinct periods revealed that the global Moran’s I indices for 2013 and 2023 were 0.632 and 0.522, respectively. These values denote a positive spatial correlation in SOC concentrations, suggesting that SOC density within a given area positively affects its neighboring SOC density. The |Z| values of 14.75 and 7.80 for each year were both above the 1.96 threshold, with corresponding p-values of 0.001, confirming statistically significant spatial clustering of SOC density. Following Moran’s I index spatial autocorrelation analysis, the evidence of a strong positive spatial autocorrelation justifies the application of Kriging interpolation as a suitable method for predicting SOC distribution.

#### 3.2.2 Semi-variogram.

**Fig 5:**
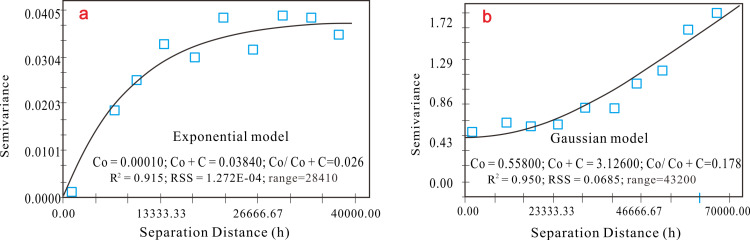
Semi-variogram for SOC data in 2013 (a) and SOC data in 2023 (b).

The semi-variogram models for SOC content in the respective years of 2013 (R^2^ = 0.915, RSS = 1.72E-04) and 2023 (R^2^ = 0.950, RSS = 0.0685) demonstrated robust fitting results, indicative of a strong regression alignment with the SOC data. Notably, the optimal fitting models differed between the two periods; with the index model providing the best fit in 2013 and the Gaussian model fitting better in 2023 (Fig 5). This shift may indicate a change in the spatial variability structure of SOC, possibly driven by changes in land management practices or environmental factors over time.

The nugget ratio C0/ (C0 + C), an indicator of SOC spatial dependence [23], was calculated as 0.026 in 2013 and 0.178 in 2023. These values underscore the strong spatial dependence of SOC, suggesting that it is predominantly influenced by structural soil factors such as soil parent material, climate, and hydrology. The notable increase in the nugget ratio from 0.026 to 0.178 signifies a transformation in the spatial variability structure of the region in two distinct temporal snapshots. Specifically, an elevated nugget ratio indicated that the nugget effect (C0) constituted a more significant proportion of the total variation (C0 + C), implying an increase in the spatial heterogeneity of SOC content.

#### 3.2.3 Spatial distribution and spatiotemporal variation of SOC.

**Fig 6:**
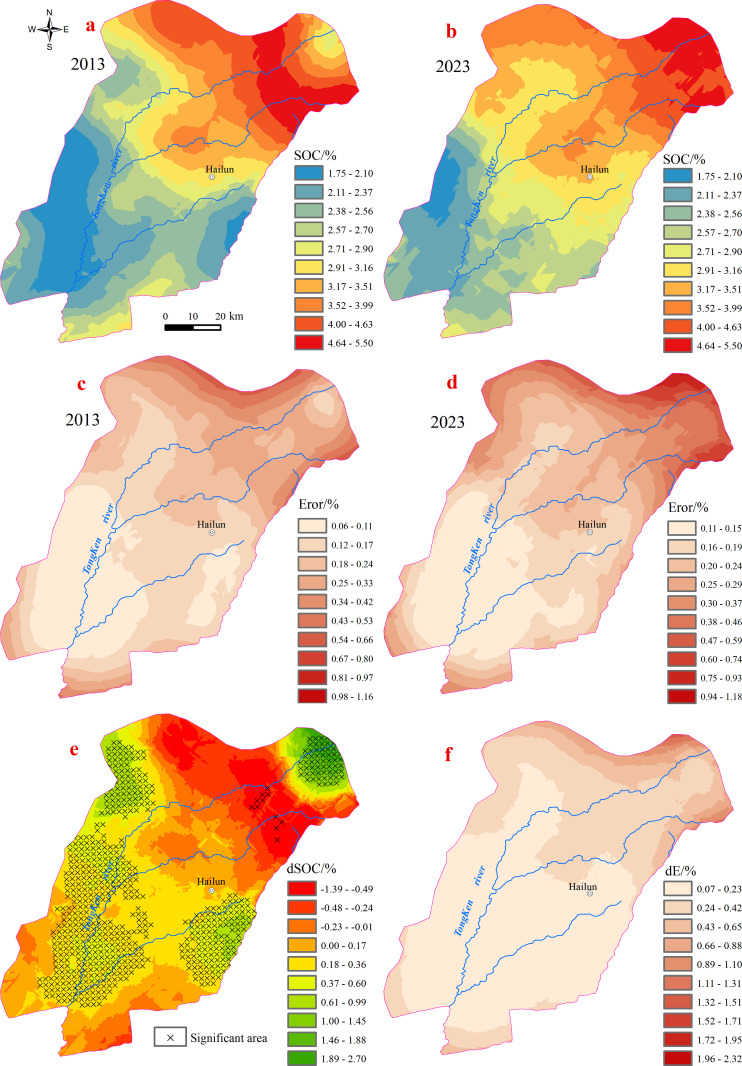
Spatial distribution of SOC content in 2013 (a) and 2023 (b), along with the corresponding standard error distributions in 2013 (c) and 2023 (d), and the spatial distribution of SOC variation map (e) and the standard error distribution of SOC variation values (f).

Figs 6(a) and 6(b) illustrate that the spatial distribution patterns of SOC in 2013 and 2023 are largely consistent. The northeastern region exhibited relatively high SOC levels, generally exceeding 4%, whereas the southwestern region had comparatively low SOC concentrations, with most areas registering below 2%. These spatial patterns reflect the underlying environmental conditions, such as precipitation and soil type, which influence the distribution of SOC.

Fig 6(e), in particular, reveals significant spatial heterogeneity in SOC changes over the 10-year period. The map shows that the northeastern hilly zone and the central to western sections of the study area experienced SOC increases, while a localized area in the northeast exhibited a notable decrease in SOC content. This suggests that while certain regions benefited from favorable land management practices, such as conservation tillage or increased vegetation cover, others may have been affected by factors like soil erosion, land use change, or climate variation, which could have led to SOC depletion.

Upon conducting significance tests on the SOC change values, it was determined that the increase in SOC content in the growing regions was statistically significant (p < 0.05), confirming that the increase in SOC in these areas is not due to random variation. However, the decrease in SOC content in the declining regions was not statistically significant (p > 0.05), suggesting that the decline in these areas could be attributed to factors that are not directly related to land management practices, such as natural climatic variability or temporary disturbances.

In summary, Fig 6(e) provides a visual representation of the spatial heterogeneity of SOC changes, supporting the conclusion that SOC dynamics in the study area are influenced by both environmental and anthropogenic factors. As shown in the figure, the SOC increase is highest in the croplands of the northeastern mountainous area, while there is no significant change in the SOC in the transitional zone between the mountains and the plains. In contrast, SOC levels in croplands of the plain areas generally show an increase. This figure highlights the importance of understanding local variations in SOC changes for targeted soil management strategies that could mitigate carbon losses and enhance soil carbon sequestration across the region.

### 3.3 Analysis result of SOC change driving factors

#### 3.3.1 Random Forest (RF) model.

**Fig 7:**
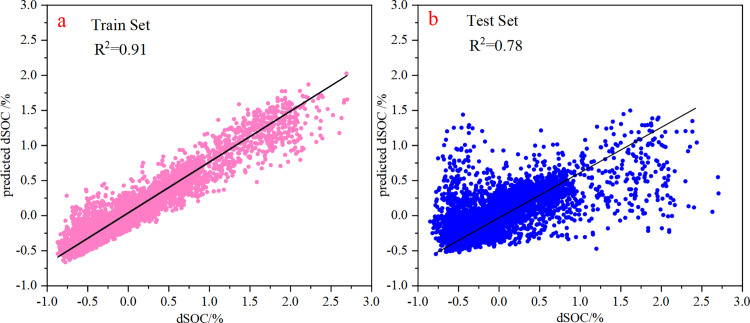
Performance of the random forest models in predicting SOC of train set (a) and test set (b).

**Fig 8:**
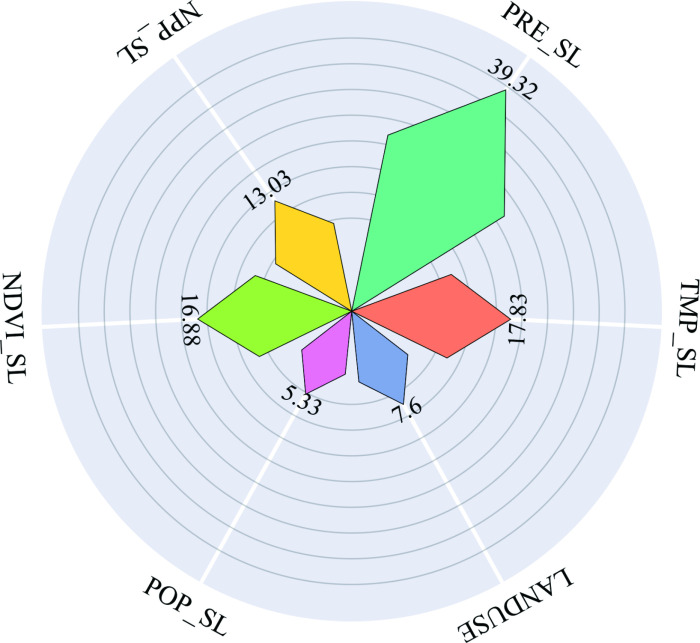
The importance ranking chart of influencing factors with the number representing the percentage of significance.

The Random Forest model demonstrated strong predictive capability, with R² values of 0.91 and 0.78 for the training and test datasets, respectively (Fig 7). These findings indicate that the RF model exhibits strong predictive capabilities. The importance of the factors affecting dSOC is shown in Fig 8. Notably, climate change emerged as the most influential factor on SOC dynamics, with PRE_SL accounting for a relative importance of 39.32%, followed by TMP_SL at 17.83%. Additionally, NDVI_SL (16.88%) and NPP_SL (13.03%) significantly contributed to dSOC variations. Although LANDUSE demonstrated relatively lower importance than the other factors, it still exerted an impact on SOC changes within the studied context. Conversely, POP_SL had minimal influence on SOC dynamics. These findings emphasize the need for integrated land management approaches that consider both climatic and land use factors to enhance SOC sequestration.

#### 3.3.2 Spearman correlation.

**Fig 9:**
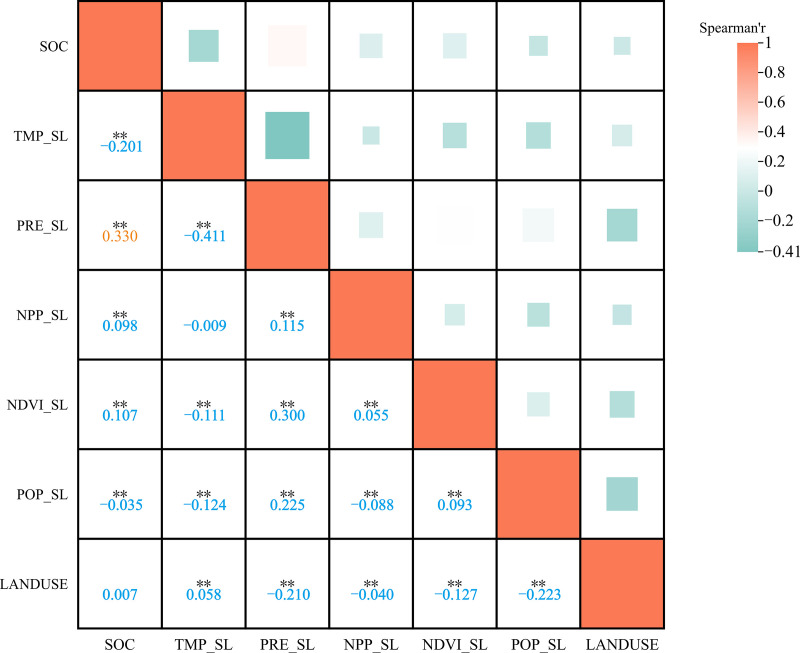
Spearman correlation between drivers and SOC variation.

**Fig 10:**
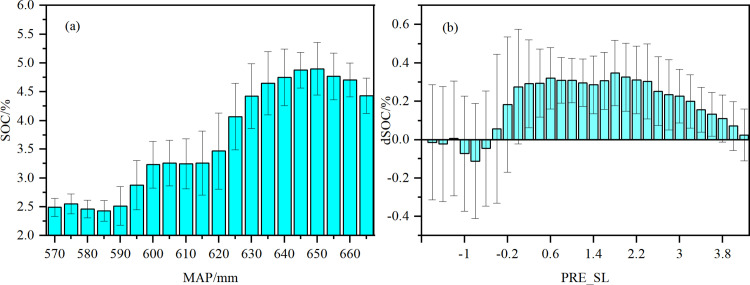
(a) Relationship between annual average precipitation (MAP) and SOC content and (b) relationship between annual precipitation change slope (PRE_SL) and SOC variation (dSOC).

**Fig 11:**
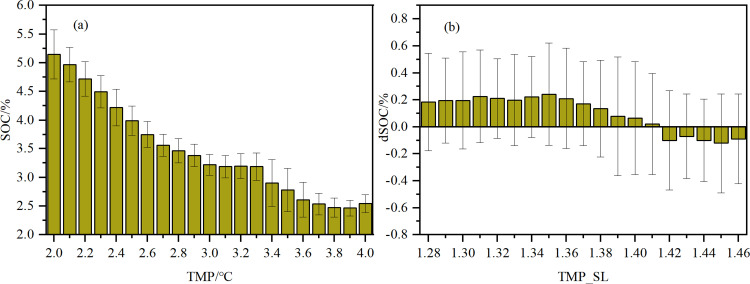
(a) Relationship between annual average temperature (TMP) and SOC content and (b) relationship between annual temperature change slope (TMP_SL) and SOC variation (dSOC).

Spearman’s correlation analysis revealed significant correlations between SOC variations (dSOC) and various environmental factors (Fig 9). A significant positive linear correlation was observed between dSOC and PRE_SL (r=0.330, P<0.01), whereas a significant negative linear correlation was observed between dSOC and TMP_SL (r=-0.201, P<0.01). Additionally, dSOC was weakly positive correlated with NDVI_SL and NPP_SL (r=0.107, P<0.01; r=0.098, P<0.01). Moreover, we also observed significant positive correlations between NDVI_SL and NPP_SL with changes in rainfall (r=0.300, P<0.01; r=0.115, P<0.01). The spatial distribution of SOC content was strongly influenced by precipitation (Fig 10), with areas receiving higher rainfall showing an increase in SOC content over time, while areas with negative precipitation trends showed decreases in SOC content. The negative correlation between SOC content and temperature (Fig 11) suggests that rising temperatures may limit SOC accumulation, particularly in regions where temperature increases surpass a certain threshold.

## 4. Discussion

### 4.1 Effects of climate change on SOC dynamic

In the past decade, the study area has shown significant changes in temperature and precipitation, and both the RF model and the correlation analyses indicate a strong correlation between these variables and the changes in SOC content.

A significant negative correlation between SOC content and temperature change was identified (Fig 9), consistent with many previous understandings[5]. Several mechanisms have been proposed to explain this phenomenon. First, elevated temperatures can enhance soil microorganism activity, thereby accelerating the decomposition rate of organic matter [32,33]. As microorganisms play a crucial role in SOC mineralization, heightened microbial activity may result in a greater release of SOC as CO_2_, consequently reducing organic carbon stocks within the soil [34]. Additionally, rising temperatures could potentially alter soil water status under drought conditions and impede plant productivity and microbial activity, thus affecting the balance between SOC input and output [35]. In forest ecosystems, the impact of rising temperatures on SOC may be positive. A decade-long soil warming experiment conducted in temperate broadleaf forests suggests that due to the instability and limited size of the forest soil carbon pool, the response of soil to temperature increases is relatively small and short-lived [36,37]. In contrast, higher temperatures generally extend the growing season and enhance photosynthetic efficiency, thereby promoting the accumulation of plant biomass. A study in northern Sweden’s spruce forests demonstrated a significant increase (over 50%) in tree stem growth under warming treatments [38]. This phenomenon suggests that elevated temperatures may enhance the input of SOC by promoting increased forest vegetation litter and root exudates [39]. Moreover, higher temperatures could also stimulate an increase in belowground biomass (such as roots), which, upon decomposition, contributes significantly to SOC pools [40,41]. This may be one of the factors driving the increase in SOC in forested areas within this study region.

Precipitation plays a crucial role in SOC accumulation by enhancing soil water availability, which simultaneously supports plant productivity and microbial-driven carbon turnover, as demonstrated by the strong positive correlation between rainfall and SOC dynamics in the RF model (Figs 8 and 9). First, elevated rainfall enhances soil water availability, directly promoting plant photosynthesis and biomass production [42]. This increases organic carbon input via plant residues (e.g., straw incorporation) and root exudates [27], as evidenced by our observed correlations between rainfall variability and NPP/NDVI trends. Second, moisture-activated microbial communities [43] regulate carbon turnover efficiency by balancing mineralization and stabilization processes during organic matter decomposition [44,45]. For instance, ^13^C tracing studies in China’s Loess Plateau demonstrated that rainfall-driven moisture increases elevate CO2 fixation rates by regulating microbial carbon flux [46]. Critically, recent analyses of northeastern China’s black soils further confirm that moisture is the dominant factor controlling topsoil (0–20 cm) SOC content [47], underscoring its overarching role in terrestrial carbon cycling.

Precipitation emerged as the most influential variable affecting SOC in the RF model, showing a strong positive correlation with changes in SOC content (Figs 8 and 9). Increased precipitation potentially contributes to SOC accumulation through two mechanisms. First, it enhances soil water availability, which in turn promotes plant photosynthesis and biomass production [42]. This leads to greater amounts of plant and root residues entering the soil through straw incorporation and other pathways, serving as important sources of organic carbon input [27]. This process is further supported by our findings, which show positive correlations between changes in rainfall and both NPP and NDVI, as previously mentioned. Second, increased precipitation elevates soil moisture levels that could further enhance the metabolic activity and biomass of soil microorganisms [43], facilitating rapid decomposition of organic materials such as straw residues, ultimately leading to an increase in SOC storage. Numerous experimental studies have demonstrated that soil moisture influences the mineralization rate of crop residues [44,45]. For instance, in China’s Loess Plateau region, researchers have employed techniques such as ^13^C labeling, metagenomic metabolic pathway analysis, and microbial community analysis to trace carbon flux during the CO_2_ fixation process. They discovered a positive correlation between rainfall and soil CO_2_ fixation rate [46]. Recent studies have also indicated that soil moisture is the most important factor influencing the SOC content in the top 0–20 cm layer of the black soil region in northeastern China[47].

Previous studies have primarily focused on the dynamics of SOC in the Northeast Black Soil region under the background of global warming, often emphasizing temperature as a key factor driving SOC changes[4,8]. Many of these studies conclude that rising temperatures accelerate SOC mineralization, leading to a decrease in SOC content. For instance, Xia et al. [48] revealed that temperature increases was the predominant factor contributing to the loss of SOC in the Song-nen Plain of Northeast China between the 1980s and 2005. In contrast, the positive effects of increased rainfall on SOC accumulation have also been well-documented, but these studies are predominantly spatial in nature[49–51]. Fewer studies have investigated how temporal variations in rainfall influence SOC dynamics, particularly in agricultural fields of Northeast China.

Our study builds on and extends previous research by providing empirical evidence of the interactive effects of temperature and rainfall on SOC dynamics. While previous studies have often considered these factors separately, our integrated approach reveals that the combined influence of climate variables and land management practices can lead to unexpected outcomes. Specifically, while rising temperatures may lead to SOC losses, these may be partially offset by the positive effects of increased rainfall and improved soil management practices in the farmland. This result emphasizes the need for region-specific studies that consider the unique interplay of local climate conditions and management practices.

### 4.2 Role of vegetation indices in SOC dynamic

The effect of vegetation indices, such as NDVI and NPP, on SOC dynamics shows a notable correlation with SOC changes (Figs 8 and 9), generally exhibiting a positive relationship. As remote sensing indicators of vegetation growth and coverage, NDVI typically correlates with plant biomass, while NPP reflects the capacity of plant communities to sequester carbon through photosynthesis over a specific time period. Numerous studies conducted in forested areas and grasslands have highlighted the effectiveness of NDVI and NPP in predicting SOC content [52,53]. A study in Liaoning Province of Northeast China demonstrated that NDVI was the most effective environmental variable for predicting SOC stocks in forest ecosystems [36].

In agricultural systems, however, the correlation between vegetation indices and SOC is often weaker due to the complex dynamics of agricultural practices [23,54]. Agricultural activities can influence both soil and vegetation in ways that weaken the direct relationship between vegetation indices and SOC [55]. For example, tillage disrupts soil structure and reduces organic matter accumulation at the soil surface, while plant growth is influenced by factors such as crop rotation, fertilization practices, and other agricultural management measures [56]. These factors can reduce the strength of the correlation between NDVI, NPP, and SOC. In regions with intensive agricultural production, changes in NDVI and NPP may not fully reflect variations in SOC [57]. Nonetheless, vegetation indices like NDVI and NPP can still provide some indication of the potential for SOC accumulation. Importantly, vegetation indices such as NDVI and NPP continue to offer valuable insights into the potential for SOC accumulation. For instance, a study in the farmland of northeast China found that, although NDVI and NPP contributed less to the spatial distribution of SOC [23], areas with higher NDVI values generally corresponded with higher biomass and greater SOC storage potential. This suggests that, despite the interference from agricultural practices, the underlying growth of vegetation remains an important factor influencing SOC dynamics.

In our study area, both NDVI and NPP exhibited an upward trend, indicating favorable conditions for plant growth and increasing agricultural productivity. From 1999 to 2019, the total grain production in the Northeast region increased from 85 million tons to 165.74 million tons [58]. Although the correlation between NDVI, NPP, and SOC was not particularly strong, the positive relationship between crop yields and SOC underscores the role of plant productivity in organic carbon accumulation. This suggests that, while vegetation indices such as NDVI and NPP may not directly quantify SOC at the regional scale, they are still indirectly linked to SOC dynamics through their association with plant productivity and the potential for organic carbon input into the soil. Therefore, improving crop productivity through better vegetation management could serve as a key strategy for enhancing SOC storage in agricultural landscapes[57]. This finding contrasts with earlier studies that emphasized a weaker direct correlation between vegetation indices and SOC [23], highlighting the importance of productivity-driven organic carbon inputs in promoting SOC accumulation.

### 4.3 Effects of soil management on SOC dynamics in forest and cropland

Soil management has a significant impact on SOC content [16,17,20]. In the study area, forest soils exhibited significantly higher SOC levels compared to dryland soils, with forest soils showing a greater increase in SOC than croplands. However, the results from the random forest model indicated that land use type explains only a limited portion of the variation in SOC (Fig. 7), suggesting that land use may not be the primary driver of SOC change in the model. This finding contrasts with the statistical analysis of the measured samples, which shows that SOC increases more significantly in forest soils than in cropland soils (Fig. 4a). This discrepancy suggests that the carbon sequestration role of forests may be underestimated in the model.

This apparent contradiction may stem from differences in data sources. Specifically, the interpolation data used in the random forest model may not accurately reflect the actual SOC conditions in forest areas, as the number of sample points in forested regions was limited. This data limitation may have led to an underestimation of the importance of forests in carbon sequestration, while the larger number of sample points in dryland areas may have resulted in an overemphasis on SOC data from these regions during interpolation, potentially masking the true contribution of forests to SOC dynamics.

Previous studies have shown a rising trend of SOC in northeastern forest soils [36,59,60]. For example, over the past 25 years, the SOC stock has increased by 471 TgC in forest ecosystems of northeastern China [59]. This increase is not only due to forest protection policies but also to the unique ecological processes in forests. The large-scale deforestation during the 1990s, driven by growing demand for timber, which led to widespread land conversion for agriculture, urbanization, industrial development, and mining [60]. However, in the past decade, the Chinese government has recognized the importance of environmental protection and implemented a series of policies aimed at forest protection [61]. This includes a comprehensive logging ban in regions such as the Lesser Khingan Mountains within the study area, greatly restoring forest cover and significantly improving the overall ecological environment [60]. Forests have a complex root system that can penetrate deep into the soil, and the roots secrete organic substances that contribute to SOC.

The shift in farming practices in northeastern China over the past decade is another key factor contributing to increased SOC [16,20,25]. The development and utilization of the black soil region have undergone two phases: large-scale exploitation and, the implementation of conservation tillage practices since 2010 [26]. Earlier studies, mostly based on data from the second national soil survey in the 1980s, reported significant declines in SOC in northeastern cropland soils over the preceding 30 years [48]. However, in the past decade, the Chinese government has prioritized the protection of black soil, implementing the Black Soil Protection Project in northeastern China and promoting practices such as straw return and other conservation tillage [19].

Conservation tillage has been shown to significantly increase SOC by reducing soil disturbance and retaining crop residues [20,26]. A meta-analysis based on 148 agricultural trial sites in northeastern China found that under various conservation tillage measures, crop yields increased by an average of 4.53%, and SOC content increased by 8.1% compared to conventional tillage [17]. Research at the regional scale in the northeastern plains also shows an increasing trend in SOC from 2006 to 2018 [23]. This is because conservation tillage reduces mechanical soil disturbance, maintains the natural soil structure, and minimizes soil erosion and compaction, thereby improving the physical stability of the soil [62]. It also enhances soil moisture retention, reduces water and soil loss, and improves soil aeration and permeability, thus increasing water use efficiency and nutrient storage capacity [21]. For example, long-term monitoring in the black soil region has shown that straw cover improves soil moisture content, increasing water use efficiency by 7.24% [62]. As a result, the impact of increased rainfall, as discussed in the previous section, becomes more pronounced. This study also revealed that the increase in SOC in croplands within agroforestry systems in the northeastern region was greater than that observed in the plain areas, which aligns with the findings of Cardinael et al.[63]. In their study of six agroforestry systems in France, they found that in silvoarable systems, SOC stocks in tree rows were significantly higher than those in inter-rows and control plots. This was primarily attributed to organic matter inputs from both the trees and the herbaceous vegetation in the tree rows. A meta-analysis further supports this, showing that SOC in agroforestry systems is influenced by factors such as abundant and diverse carbon sources (e.g., litterfall, straw, and root biomass), improved soil properties (e.g., reduced erosion and increased nutrient content), and enhanced microbial activity [64]. Previous studies have highlighted the critical role of land use change in driving SOC dynamics in the black soil region of northeast China [8]. In the study area, the predominant form of land use change is the conversion of dryland to paddy fields. The persistent waterlogging conditions in paddy fields alter soil oxygen availability, creating anaerobic environments that slow microbial decomposition rates and reduce organic carbon mineralization rates [65]. Nevertheless, since paddy fields account for only about 3% of the total area in the study region, the overall impact of land use change on SOC is relatively limited. However, the conversion of drylands to paddy fields in localized areas may still have some effect on SOC accumulation and release.

### 4.4 Practical applications

The findings of this study offer practical implications by providing specific recommendations for soil management practices and policy frameworks. Precipitation plays a significant role in SOC dynamics, especially in areas with high or variable rainfall. To address this, we recommend adjusting agricultural practices in these regions based on seasonal variations in precipitation. For instance, during wetter seasons, reducing tillage frequency, increasing crop cover, or implementing conservation tillage practices can help reduce soil erosion and enhance soil organic carbon stocks. In addition, areas with variable rainfall should adopt optimized water management practices to better maintain soil moisture and the stability of organic carbon.

This study affirms the positive effect of conservation tillage on SOC in cropland, while also highlighting that agroforestry systems lead to even greater increases in SOC. Therefore, we recommend promoting the transition from monoculture farming to agroforestry systems in suitable areas [64], while continuing to scale up conservation tillage practices. When developing relevant policies, governments should carefully consider the ecological characteristics of different regions and support sustainable land management and conservation agriculture practices to promote long-term carbon sequestration.

### 4.5 Limitations and implications

Conservation tillage practices such as straw incorporation have been implemented in this region and could potentially exert a significant influence on SOC dynamics. However, the lack of data pertaining to straw incorporation methods and quantities hinders quantification of their impact on SOC. Additionally, this study postulated that increased rainfall may enhance microbial activity and accelerate straw decomposition rates, thereby augmenting SOC content; however, direct measurements of microbial activity and straw decomposition rates were not conducted. Further experimental evidence is required to substantiate this explanation. Nevertheless, the results of this study highlight the critical role of rainfall variability in the organic carbon cycle, and provide valuable data to support the development of land management strategies aimed at adapting to climate change and enhancing soil carbon sequestration. Additionally, this study solely employed Kriging interpolation to estimate the spatial variation of SOC content, which may lead to an incomplete understanding of spatial variation. To gain a more comprehensive understanding of the spatial variation patterns of SOC content, future studies could consider employing other spatial analysis methods such as trend surface analysis, geographically weighted regression, or other geostatistical methods, or combining multiple approaches for a more thorough analysis.

The overall increase in SOC in the study area resulted from the positive impact of incorporating straw into the field and the rise in rainfall, as well as the negative influence of elevated temperatures. In future climate scenarios, uncertainties regarding precipitation patterns and temperature projections amplify the challenges associated with predicting SOC trends in SOC. To enhance the accuracy of forecasting and managing changes in SOC, it is necessary to gain insight into how these factors individually and collectively affect the process of carbon accumulation. This may entail integrating data from multiple sources and adopting more advanced models to make precise predictions about future trends in SOC within the context of global warming and potential alterations in precipitation patterns.

## 5. Conclusions

In this study, the impacts of climate change and land management on SOC dynamics in northeastern China were explored. The results showed a significant increase in both SOC content and density from 2013 to 2023 (p<0.01), indicating that the study area functioned as a carbon sink during this period. Temperature and precipitation were found to significantly influence SOC content, with elevated temperatures generally decreasing SOC due to accelerated organic matter decomposition, while increased precipitation promoted SOC accumulation by enhancing plant productivity and soil moisture. Forest soils exhibited higher SOC levels, with recent restoration efforts contributing to SOC increases in these areas. Agricultural practices, particularly conservation tillage, also played a positive role in SOC accumulation in croplands.

The findings of this study have practical implications for land management and SOC policies, emphasizing the importance of integrating climate factors and sustainable land practices to maintain or increase SOC stocks. Future research should further investigate the long-term impacts of specific management practices on SOC across different ecosystems, as well as the role of local soil properties and microbial activity in SOC dynamics. These studies would contribute to a deeper understanding of how SOC responds to climate change and land use.
